# Membrane closure in stress induced-autophagosome formation

**DOI:** 10.15698/cst2018.06.138

**Published:** 2018-06-11

**Authors:** Eiji Morita

**Affiliations:** 1 Department of Biochemistry and Molecular Biology, Faculty of Agriculture and Life Science, Hirosaki University.

**Keywords:** alternative autophagy, membrane closure, DRAM1

Autophagy (macroautophagy) is a bulk degradation system that sequesters cytoplasmic components by generating double-layered isolation membranes and delivering these isolated contents to the lysosomes 1. Though this pathway was originally identified as a system essential for the replenishment of nutritional source by the self-degradation of cellular components, later it has drawn attention as a pathway that is required for the clearance of unnecessary constituents such as damaged organelles, aggregated proteins, and invasive pathogens. In yeast genetics, at least 18 autophagy-related (ATG) genes were identified as the factors required for the autophagosome formation. Recent biochemical analyses revealed the roles of these ATG gene products in autophagosome membrane biogenesis 1.

Autophagy is one of the cellular stress responses that is induced by various types of environmental conditions such as starvation, oxidative stress, heat shock, and pathogenic infection 2. Cells have mechanisms and processes to maintain homeostasis in order to survive during various unfavorable conditions, i.e. by removing damaged organelles and other cellular contents through stress-induced autophagy. Therefore, a deficiency of autophagic machinery causes several diseases. It is well-known that the risk of Crohn's disease occurrence increases in the presence of ATG16L1 mutation 3. Genetic polymorphisms of ATG5 are associated with asthma and enhanced risk of systemic lupus erythematosus 4. The deletion of Beclin1 gene is associated with risk and prognosis of several human cancers 5. Autosomal recessive and sporadic early-onset Parkinson's diseases are associated with mutations in parkin and PTEN-induced putative kinase 1 (PINK1), genes that are required for the selective autophagic degradation of mitochondria so-called as mitophagy 6.

DNA damage is another well-known stress condition that induces autophagy 7. In cells irradiated with ultra violet or ionizing radiation, or treated with DNA damage-inducing chemicals, the number of autophagosomal double-membrane structures is increased in the cytoplasm. DNA damage-induced autophagy is one of the cytoprotective systems as it removes damaged components that are hazardous to normal cell machinery and in turn protect the cell from cell death 8. Therefore, the deficiency of autophagy machinery causes cells to be more sensitive towards DNA damage-induced apoptosis. This response is known to be dependent on p53 activation and the transactivated downstream factor of p53 is considered a key molecule that regulates the induction of autophagy 9. Recently, protein phosphatase Mg^2+/^Mn^2+^ dependent 1D (PPM1D) which belongs to Ser/Thr protein phosphatases that are transactivated by p53 was identified as a factor that dephosphorylates serine-637 of unc-51 like autophagy activating kinase (ULK1) 10. This study suggested a model that the aforementioned dephosphorylation event might be a trigger for the initiation of autophagosome biogenesis. ULK1 is a subunit of the ATG1-complex that functions at the most upstream position in ATG signaling and the dephosphorylation of this complex is well-known to be essential for the induction of autophagy during other conditions such as starvation. It suggests that DNA damage-induced autophagy could be regulated by the same machinery as that in autophagy induced by other stresses. However, Nishida *et al*. reported that a type of autophagy induced by DNA damage might be driven by various factors that were not previously identified during initial yeast genetic screenings 11,12. They found that a few types of autophagosomes are formed independent of ATG5 and ATG7 function. ATG5 and ATG7 are well-known factors that participate in the phosphatidylethanolamine (PE) conjugation with micro-tubule-associated protein 1 light chain 3 (LC3). In ATG5 or ATG7 knockout cells, the lipid modification of LC3 and its consequence i.e. LC3 puncta formation are never observed. Therefore, these events could be the signature steps of autophagosome formation and the monitoring of LC3-lipid modification is a widely used method to detect autophagosome induction or accumulation 1. Nishida *et al*. observed the accumulation of autophagic isolation membrane in ATG5 or ATG7-deficient cells during a few conditions. Therefore, they concluded that a mechanism of autophagy is independent of the ATG-lipid conjugation system and named this type of autophagy as ‘alternative autophagy’ 11. Similar observations were reported by several other groups. Fujita *et al*. reported that autophagic membranes were accumulated in the cells that expressed ATG4B C74A mutant that efficiently blocks ATG conjugation reaction 13. Itakura *et al*. reported that the isolation membrane was observed in carbonyl cyanide m-chlorophenylhydrazone (CCCP)-induced parkin-dependent mitophagy even in ATG3-deficient cells 14. Tsuboyama *et al*. reported that syntaxin 17-positive autophagosomes were observed in ATG3 deficient cells 15. The phenomena observed in these reports were mostly similar but the mechanism of alternative autophagy is distinct compared to the aforementioned phenomena. Nagata *et al*. demonstrated that syntaxin 17 (a marker that is recruited during the conventional starvation-induced autophagosome formation) does not localize at the DNA damage-induced autophagosome/autolysosome 16. In addition to the recruitment of syntaxin 17, there appear to be several variations in this mechanism 12. However, till date, sufficient information is unavailable to completely understand this system.

In this study, Nagata *et al*. reported the role of damage-regulated autophagy modulator 1 (DRAM1) in the alternative autophagy 16. DRAM1 was originally identified as a factor that is transactivated by p53. The cells depleted of this gene become more sensitive to apoptosis under the DNA damage-induced conditions 9. As the DRAM1 gene produces transmembrane lysosomal proteins, the depletion and expression of DRAM1 impairs and induces autophagosome formation, respectively. Therefore, DRAM1 might be a cytoprotective factor that prevents cell death through the induction of autophagy under the genotoxic stress condition. Nagata *et al*. revealed that these phenomena were observed in ATG5-deficient cells, suggesting that DRAM1 might function during autophagosome formation in the alternative pathway. Moreover, DRAM1 localizes at the LC3-positive puncta in wild type cells, indicating that this factor functions in the alternative as well as conventional pathway 16.

Interestingly, in DRAM1-depleted cells, autophagosome maturation gets arrested exactly prior to the membrane closure step. During the autophagosome formation, double-membranes must be completely enclosed and inner and outer membranes must be separated. This event is required for the sequestration of contents in order to deliver them to the lysosome. Nagata *et al*. observed that several abnormally swollen isolation membranes were accumulated in DRAM1-depleted cells, by performing electron microscopic analysis, and suggested that DRAM1 might play a role in the membrane closure step of autophagosome formation 16.

Molecular mechanisms underlying membrane closure in autophagosome formation are considered to be related to multivesicular body (MVB) biogenesis 17. In the MVB formation, endosomal sorting complex required for transport (ESCRT) participates in the formation of inter lumenal vesicles (ILVs) from endosomal membrane. ESCRT factors are known to function during the membrane pinching-off stage of ILV formation by forming a circular array inside the budding neck and creating pressure for membrane scission. Membrane closure step is mostly a similar event in autophagosome formation as well as ILV formation as autophagosome formation is merely the formation of a single large ILV from the isolation membrane 18. In theory, during the closure of double membrane, the membrane topology should be made of a suitable substrate in order to be sealed by the ESCRT factors. Till date, there is no critical evidence that ESCRT factors are involved in the membrane closure step of autophagosome formation. However, it would be not surprising that ESCRT is involved in this step and it is highly possible that DRAM1 be involved in this step along with ESCRT.

During the autophagosome formation, the completion of membrane closure step might be an important signal for its fusion with the lysosome. Though the precise molecular machinery that participates in the completion of this event that leads to the lysosome fusion is unclear, syntaxin 17 is identified as a factor that gets recruited to the autophagosome membrane only after the membrane closure and functions as a SNARE during lysosome fusion in starvation-induced conventional autophagy 19. It is very important to understand the regulatory mechanism of these events in alternative autophagy.
